# Interactional synchrony in chimpanzees: Examination through a finger-tapping experiment

**DOI:** 10.1038/srep10218

**Published:** 2015-05-11

**Authors:** Lira Yu, Masaki Tomonaga

**Affiliations:** 1 Primate Research Institute, Kyoto University; 2 Japan Society for the Promotion of Science

## Abstract

Humans often unconsciously coordinate behaviour with that of others in daily life. This interpersonal coordination, including mimicry and interactional synchrony, has been suggested to play a fundamental role in social interaction. If this coordinative behavior is socially adaptive, it may be shared with other highly social animal species. The current study targeted chimpanzees, which phylogenetically are the closest living relatives of humans and live in complex social groups, and examined whether interactional synchrony would emerge in pairs of chimpanzees when auditory information about a partner’s movement was provided. A finger-tapping task was introduced via touch panels to elicit repetitive and rhythmic movement from each chimpanzee. We found that one of four chimpanzees produced significant changes in both tapping tempo and timing of the tapping relative to its partner’s tap when auditory sounds were provided. Although the current results may have limitations in generalizing to chimpanzees as a species, we suggest that a finger-tapping task is one potential method to investigate interactional synchrony in chimpanzees under a laboratory setup.

Humans often unconsciously coordinate their movements with those of others^1^. This interpersonal coordination includes two distinctive behaviours: mimicry and interactional synchrony^2^. Mimicry involves matching a type of behaviour, such as posture, body configuration, or facial expression, to another individual. Interactional synchrony, in contrast, is matching the timing of a movement, such as synchronous hand-clapping in a concert hall or stride-matching while walking with other individuals. Experimental studies have further shown that mimicry and interactional synchrony occur under automatic modulation in humans^3^,^4^,^5^,^6^,^7^.

Despite being common in humans, the function of interpersonal coordination remains a topic of investigation. Previous studies have shown that mimicry and interactional synchrony have positive social consequences in humans: when we are mimicked by or synchronized to others, we tend to show increased affiliation, rapport and feelings of empathy towards them^1^,^8^, and behave more prosocially in general^9^. These findings suggest that interpersonal coordination, both mimicry and interactional synchrony, plays an important role in maintaining positive social relationships among humans.

Interestingly, one study showed that other highly social non-human primate species share an adaptive function of interpersonal coordination with humans^10^. Capuchin monkeys (*Cebus capucinus*), which are a highly social primate species, preferred humans who imitated their behaviours over non-imitators. This finding suggests that mimicry may also play a positive social role in their actual interactions. However, it remains unclear whether non-human primates coordinate with conspecifics in a similar manner as that shown between capuchins and humans. Previous studies on mimicry have shown that non-human primates produce contagious yawning^11^,^12^ and neonatal facial mimicry^13^,^14^,^15^, which suggest that humans are not unique in their ability to mimic other’s facial expressions in a spontaneous manner.

Relative to the studies of mimicry, there are few studies of interactional synchrony in non-human primates. To date, two experimental studies have shown that mechanisms of spontaneous synchronization to external stimuli exist in non-human primates. Nagasaka, Chao, Hasegawa, Notoya & Fujii (2013) reported that Japanese macaques (*Macaca fuscata*) synchronized their arm movements with partner monkeys that were facing them^16^. In a further test, they demonstrated that visual information of the other’s movement was required to produce interactional synchrony, while auditory information of the other’s movement had little influence. In contrast to the study of the macaques, Hattori, Tomonaga & Matsuzawa (2013) reported that a chimpanzee (*Pan troglodytes*) could synchronize to auditory sounds specifically when the sounds were isochronous and close to the chimpanzee’s preferred tempo^17^. These two studies indicate that chimpanzees and macaques may have different sensitivities to auditory information when producing synchronized movement^18^. However, little is known about whether chimpanzees are sensitive to auditory sounds of an irregular and fluctuating tempo that are produced by other conspecifics.

In the current study, we investigated whether interactional synchrony occurs in pairs of chimpanzees (*Pan troglodytes*) when they are sitting side by side and auditory information about the other’s movement is provided. Studies on chimpanzees are crucially important for understanding the evolutionary origins of interactional synchrony in humans, because they are phylogenetically the closest living relatives to humans. We introduced a finger-tapping task, which is extensively used for exploring sensorimotor synchronization in humans^19^. The task was adapted to touch screen monitors, which facilitated repetitive and rhythmic tapping movements and enabled observation of interactional synchrony between chimpanzees in the current laboratory setup. The chimpanzees performed the task under two different auditory conditions: with and without auditory sounds.

## Results

**Figure 1:**
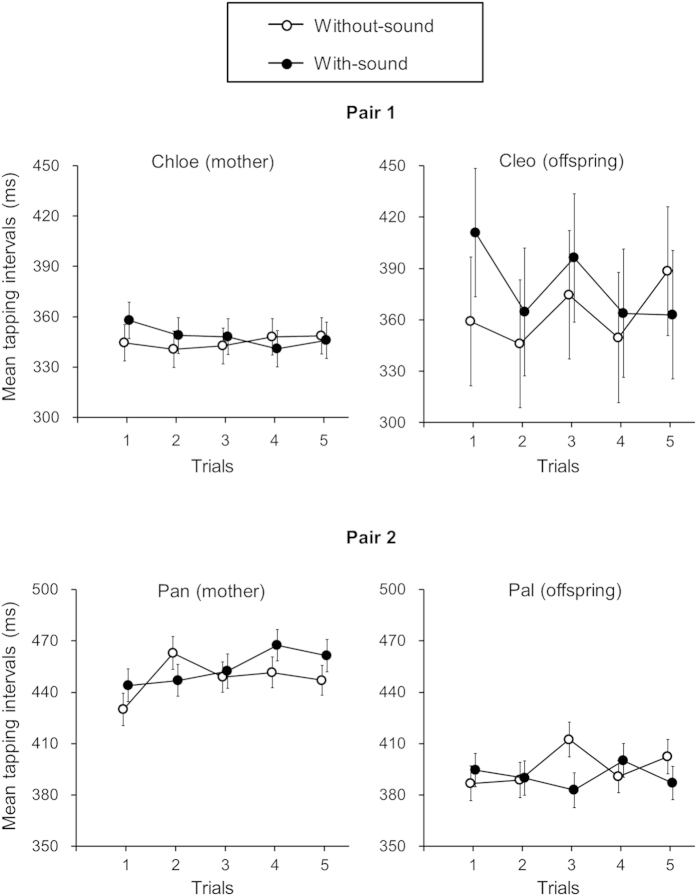
Mean tapping intervals across five trials under two auditory conditions for each chimpanzee. Error bars represent 95% confidence intervals of the mean.

We first examined the effect of auditory sounds on the chimpanzees’ tapping movement. If auditory sounds interfered with the chimpanzees’ tapping movements, the tapping behaviour in the with-sound condition would differ from the without-sound condition. To test this hypothesis, the mean tapping interval, which is an inverse form of tempo, was compared between the without-sound and with-sound conditions. Some of the tapping intervals from a ‘miss-touching’ event were excluded from the data analysis. Excluded data were less than 10% for each participant and for each condition. For the statistical analysis, an effect of trial was additionally examined to see how tapping intervals change as time progresses. We found that all four chimpanzees produced distinctive patterns of tapping intervals across trials in the with-sound condition compared to the without-sound condition. There was significant interaction between condition and trial: Chloe, F(4,1612) = 4.427, p = 0.001; Cleo, F(4,1567) = 4.408, p = 0.002; Pan, F(4,1401) = 5.087, p < 0.001; Pal, F(4,1752) = 5.637, p < 0.001 (Fig. 1).

**Figure 2:**
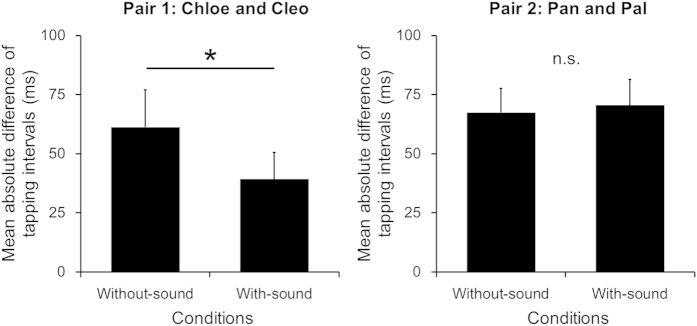
Mean absolute difference in tapping intervals under two auditory conditions for each pair of chimpanzees. Error bars represent 95% confidence intervals of the mean.

Next, we tested whether these distinctive patterns of tapping intervals in the with-sound condition were a response to auditory sounds from the partner chimpanzee. If there was an effect of auditory sounds from the partner chimpanzee, a convergence of tapping intervals would occur between the chimpanzees. To examine this hypothesis, we calculated an absolute difference in tapping intervals between the paired chimpanzees for each trial (Fig. 2). A median tapping interval was used as a value of central tendency per trial for each chimpanzee. We found that for one pair of chimpanzees, Chloe and Cleo, the mean absolute difference in tapping intervals between them was significantly smaller in the with-sound condition compared to the without-sound condition, t(29) = 2.229, p = 0.03. Another pair of chimpanzees, Pan and Pal, showed no significant changes between the without-sound and the with-sound condition, t(29) = 0.407, p = 0.685.

**Figure 3:**
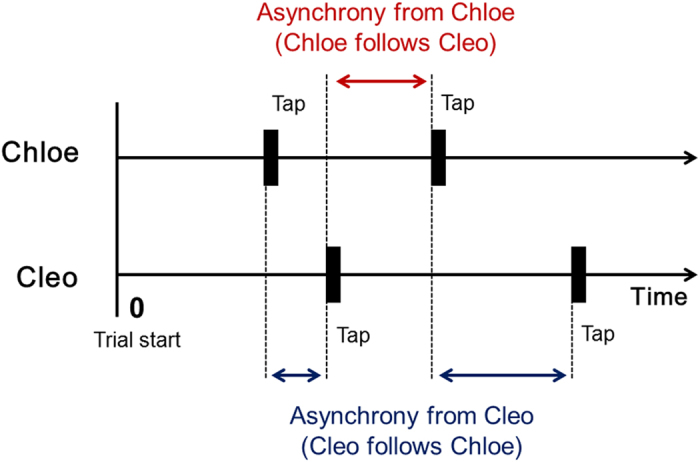
Schematic representation of asynchrony.

**Figure 4:**
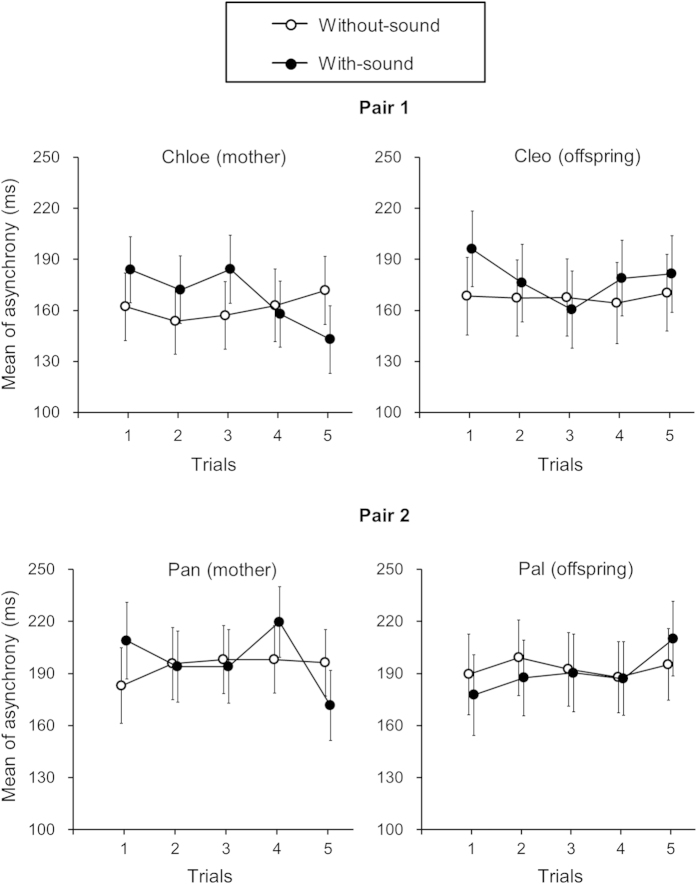
Mean asynchrony across five trials under two auditory conditions for each chimpanzee. Error bars represent 95% confidence intervals of the mean.

Finally, we examined whether tapping movements would converge in time among pairs of chimpanzees. We calculated the asynchrony (Fig. 3), which was defined as the minimal difference in timing from a partner’s tap to a chimpanzee’s own tap. Consistent with the analysis on tapping intervals, we examined this for an effect of condition and trial (Fig. 4). We found that the mean asynchrony from Chloe, that is, the difference in timing between the pair when Chloe followed her partner Cleo’s tap, became significantly smaller as the trial progressed in the with-sound condition. Chloe showed a significant interaction between condition and trial, F(4,1406) = 3.171, p = 0.013. A post hoc analysis revealed a simple main effect of trials within the with-sound condition, F(4,1406) = 3.730, p = 0.005. Multiple comparisons based on estimated marginal means with Sidak’s correction further revealed that the mean asynchrony for the 5th trial was significantly smaller than the 1st and 3rd trials (1st vs. 5th, p = 0.016; 3rd vs. 5th, p = 0.017). In the other three chimpanzees, we found no significant interaction or main effect of the condition or of the trial on asynchrony.

## Discussion

In the current study, we investigated whether interactional synchrony would occur in pairs of chimpanzees when auditory information of the partner’s movements was provided. All four chimpanzees were affected by auditory sounds; however, a convergence of tapping intervals was found only in the pair of Chloe and Cleo. This suggests that auditory sounds from partners could affect a chimpanzee’s own tapping tempo. Asynchrony analyses demonstrated that Chloe augmented her tapping movements to match those of her partner’s, particularly as the trial progressed and when auditory sounds were provided. Although the synchronization shown by Chloe was relatively weak compared to that of humans, her behaviour was consistent with previous studies showing that both the tempo and timing of movements (represented by tapping intervals and asynchrony, respectively) change when humans synchronize to musical beats or with other individuals^6^,^18^,^19^,^20^. Considered together, we demonstrated that at least one chimpanzee could produce interactional synchrony with auditory sounds from her partner.

None of the chimpanzees in the current experiment were trained to match their tapping behavior with the others. Thus, the behaviours seen in Chloe are consistent with previous studies demonstrating that non-human primates can produce spontaneous synchronization with external stimuli even without explicit training^16^,^17^.

Although this study cannot discern whether the presence of the other (partner) chimpanzee is an important factor for the emergence of interactional synchrony, we suggest that Chloe’s behaviour differed from the way in which chimpanzees coordinate with isochronous auditory sounds in non-social settings. Hattori, Tomonaga & Matsuzawa (2013) showed that a chimpanzee named Ai synchronized to isochronous auditory sounds that were close to her own preferred tempo, but not to distant or random tempos^17^. In contrast, Chloe synchronized with the auditory sounds of an irregular and distant tempo. These differences imply that the presence of a partner might facilitate interactional synchrony with a fluctuating tapping tempo generated by another individual.

Previous studies on humans demonstrated that synchronous handclapping can disappear and reappear several times during applause^20^,^21^. However, in non-human primates, the way in which interactional synchrony occurs and changes over time has not yet been revealed. To our knowledge, this is the first study showing that a chimpanzee coordinated her own rhythmic movement with the interacting partner in a more synchronous manner as time progressed. To further confirm a gradual process in interactional synchrony among chimpanzees, our future research will examine the execution of tapping movements under a prolonged duration in a laboratory setting.

Despite our results, we admit that the current study may not allow us to generalize to all chimpanzees because only one of four subjects showed a tendency toward interactional synchrony with a partner. A long history of conducting independent computer tasks^22^,^23^ while ignoring others might have overshadowed the effect of auditory sounds in the current experimental setting. Further study is needed under a new laboratory setup to exclude this plausible artefact.

In summary, the current study demonstrated that interactional synchrony can occur between chimpanzees, as observed in a single chimpanzee that augmented her tapping movements upon hearing auditory sounds to converge on her partner’s movements. Although the current study requires further evidence to suggest that chimpanzees may have the capacity to modulate their behaviours to synchronize with other conspecifics, it demonstrated that a finger-tapping task is one potential method for investigating interactional synchrony under a laboratory setting. Compared to a recent observational study showing that a chimpanzee synchronized a motor action with another chimpanzee in the context of observational learning^24^, experimental studies such as the current one can clarify cognitive processes related to behaviour, such as visual and auditory perception and attention, to understand the underlying mechanisms of interpersonal coordination.

Future studies should consider social relationships to explore what social factors may influence the intensity of interactional synchrony in chimpanzees and other animals. Although the current study focused on mother-offspring pairs, field observations have documented coordinated behaviours in male chimpanzees, such as during territorial boundary patrols^25^,^26^ and vocal chorusing^27^,^28^,^29^. To understand the adaptive significance of interactional synchrony and its relevance to empathy^39^ in chimpanzees, our future study will also investigate pairs of unrelated chimpanzees with a range of social relationships.

## Methods

### Participants

Two pairs, each consisting of a mother chimpanzee and her biological offspring (33-year-old Chloe and her 13-year-old daughter, Cleo; and 30-year-old Pan and her 13-year-old daughter, Pal) participated in the experiment. Both offspring had been successfully raised by their mothers since they were born at the Primate Research Institute, Kyoto University^22^. They lived together with nine other chimpanzees in an enriched outdoor compound with attached indoor residences^30^. All experiments were voluntary, and subjects were never deprived of water or food. All chimpanzees had extensive experience with cognitive tasks using a touch screen^23^ and some of the experiments included auditory cues as a discriminative stimulus^31^,^32^,^33^. Studies on social interactions between chimpanzees have also been conducted under various experimental setups^34^,^35^,^36^,^37^,^38^. The care and use of chimpanzees were carried out in accordance with the 3^rd^ edition of the *Guide for the Care and Use of Laboratory Primates* issued by Kyoto University Primate Research Institute in 2010. The experimental protocol was approved by the Animal Welfare and Animal Care Committee of the same institute.

### Apparatus

Experiments were conducted in an experimental booth (1.8 × 2.15 × 1.75 m) adjacent to the chimpanzees’ indoor residences. Two interconnected 17-inch LCD monitors (1280 × 1024 pixels) with a touch panel were used, and were set approximately 80 cm apart on the same side of the wall of the booth. Two universal feeders delivered a food reward to each chimpanzee. Auditory stimuli were played through a built-in speaker of the monitor. All the equipment and experimental events were controlled by a computer located outside the experimental booth.

### Procedure

A finger-tapping task was introduced to elicit repetitive and rhythmic tapping movements from each chimpanzee using a touch panel. Each trial began with the presentation of a Start button on the screen. After touching the button, the chimpanzees were required to touch a visual target (5.8 × 5.8 cm) that appeared on the left or the right side of the monitor. When the target that appeared was touched once, it disappeared with a short beep sound (55 ms, 1000 Hz, approximately 50 dB) and reappeared on the other side with 0 ms delay. After the left and the right targets were tapped alternatively several times, a trial ended with a chime, and food rewards were delivered to the chimpanzees. The inter-trial interval was 2 seconds. Each chimpanzee was individually trained to conduct the task, starting with a single tap, and then the number of required taps increased up to between 25 and 35. This training phase continued until the chimpanzees cleared a criterion of tapping without a pause of more than 3 seconds during a trial.

**Figure 5:**
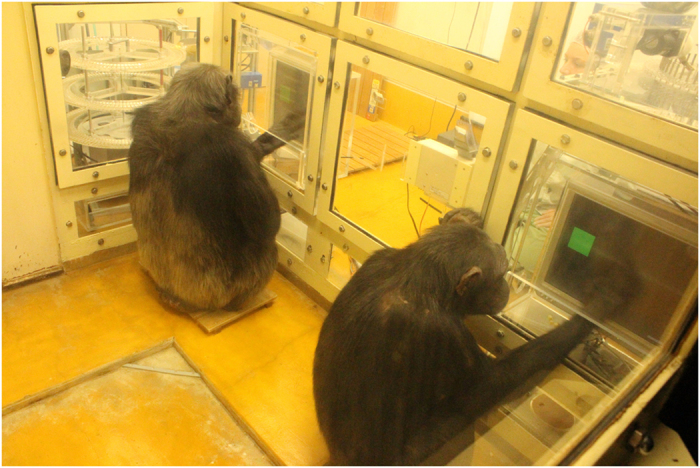
Chimpanzees (left: Chloe, right: Cleo) conducting a finger-tapping task.

During the test phase, the pairs of chimpanzees conducted a finger-tapping task simultaneously (Fig. 5). Two experimental conditions were prepared: without-sound and with-sound condition (see Supplementary Video 1). In the without-sound condition, a short beep sound corresponding to each chimpanzee’s tapping event was absent so that the sounds would not interfere with the chimpanzees’ own preferred rhythmic movement. In contrast, in the with-sound condition, each tap by the chimpanzees was accompanied by a short beep sound so that the auditory information corresponding to movement could be exchanged between chimpanzees.

In the test phase, a yoked-control procedure was used to equate the time of a trial Start and Finish between the chimpanzees. Master and yoked subject were randomly assigned on a trial basis. However, the timing of visual target presentation was not controlled by the experimenter for both chimpanzees. The target reappeared with 0 ms delay when the previous target was touched. Both chimpanzees in a pair produced the tapping movement with their own-preferred tempo across the experiment.

### Experimental design and statistical analysis

To examine whether the chimpanzees changed their tapping behavior depending on condition, 30 trials were collected for each test condition from each chimpanzee. Each trial required 25 to 35 tapping movements. Five trials were conducted per block and 6 blocks were obtained over four experimental days. ABA block design was employed to randomize the order of the two test conditions. Either an ABA or a BAB schedule was conducted in any given day.

In addition to an effect of conditions, we also examined an effect of trial number because we predicted that tapping behavior would change depending on time. We used generalized linear mixed model (implemented in SPSS Advanced Models 14.0J) for data analysis. The fixed effects were Conditions and Trials, and the random effect was Blocks (nested in trials)^40^. No multiple comparisons within-pairs or within-the four chimpanzee participants was conducted in the analysis.

## Author Contributions

L.Y. designed and conducted the experiment. L.Y. and M.T. analysed the data and discussed the results. L.Y. wrote the paper, and M.T. commented on the manuscript.

## Additional Information

**How to cite this article**: Yu, L. and Tomonaga, M. Interactional synchrony in chimpanzees: Examination through a finger-tapping experiment. *Sci. Rep.* **5**, 10218; doi: 10.1038/srep10218 (2015).

## Supplementary Material

Supplementary Information
