# Corrigendum: Interactional synchrony in chimpanzees: Examination through a finger-tapping experiment

**DOI:** 10.1038/srep14031

**Published:** 2015-09-21

**Authors:** Lira Yu, Masaki Tomonaga

Scientific Reports 5: Article number: 1021810.1038/srep10218; published online: 05112015; updated: 09212015

This Article contains the following typographical errors in the reference citations.

In the Discussion section,

“To understand the adaptive significance of interactional synchrony and its relevance to empathy^39^ in chimpanzees, our future study will also investigate pairs of unrelated chimpanzees with a range of social relationships.”

should read:

“To understand the adaptive significance of interactional synchrony and its relevance to empathy^30^ in chimpanzees, our future study will also investigate pairs of unrelated chimpanzees with a range of social relationships.”

In the Methods section under the subheading ‘Participants’,

“They lived together with nine other chimpanzees in an enriched outdoor compound with attached indoor residences^30^.”

should read:

“They lived together with nine other chimpanzees in an enriched outdoor compound with attached indoor residences^31^.”

And lastly, “All chimpanzees had extensive experience with cognitive tasks using a touch screen^23^ and some of the experiments included auditory cues as a discriminative stimulus^31,32,33^. Studies on social interactions between chimpanzees have also been conducted under various experimental setups^34,35,36,37,38^.”

should read:

“All chimpanzees had extensive experience with cognitive tasks using a touch screen^23^ and some of the experiments included auditory cues as a discriminative stimulus^32,33,34^. Studies on social interactions between chimpanzees have also been conducted under various experimental setups^35,36,37,38,39^.”

